# *In Vitro* Studies of Chromone-Tetrazoles against Pathogenic Protozoa, Bacteria, and Fungi

**DOI:** 10.3390/molecules200712436

**Published:** 2015-07-08

**Authors:** Pedro A. Cano, Alejandro Islas-Jácome, Ángeles Rangel-Serrano, Fernando Anaya-Velázquez, Felipe Padilla-Vaca, Elías Trujillo-Esquivel, Patricia Ponce-Noyola, Antonio Martínez-Richa, Rocío Gámez-Montaño

**Affiliations:** 1 Departamento de Química, División de Ciencias Naturales y Exactas, Universidad de Guanajuato, Noria Alta S/N, Col. Noria Alta, Guanajuato C.P. 36050, Gto., Mexico; E-Mails: pedroacanom@gmail.com (P.A.C.); blackheim66@gmail.com (A.I.-J.); richa@ugto.mx (A.M.-R.); 2 Departamento de Biología, División de Ciencias Naturales y Exactas, Universidad de Guanajuato, Noria Alta S/N, Col. Noria Alta, Guanajuato C.P. 36050, Gto., Mexico; E-Mails: arangel@ugto.mx (A.R.-S.); anayafe@ugto.mx (F.A.-V.); padillaf@ugto.mx (F.P.-V.); elias_tru@hotmail (E.T.-E.); poncep@ugto.mx (P.P.-N.)

**Keywords:** chromone-tetrazoles, Ugi-azide reaction, antimicrobial activity, pathogenic protozoa, pathogenic bacteria, pathogenic fungi, tropical diseases

## Abstract

*In vitro* studies to fourteen previously synthesized chromone-tetrazoles and four novel fluorine-containing analogs were conducted against pathogenic protozoan (*Entamoeba histolytica*), pathogenic bacteria (*Pseudomonas aeruginosa*, and *Staphylococcus aureus)*, and human fungal pathogens (*Sporothrix schenckii*, *Candida albicans*, and *Candida tropicalis*), which have become in a serious health problem, mainly in tropical countries.

## 1. Introduction

**Figure 1:**
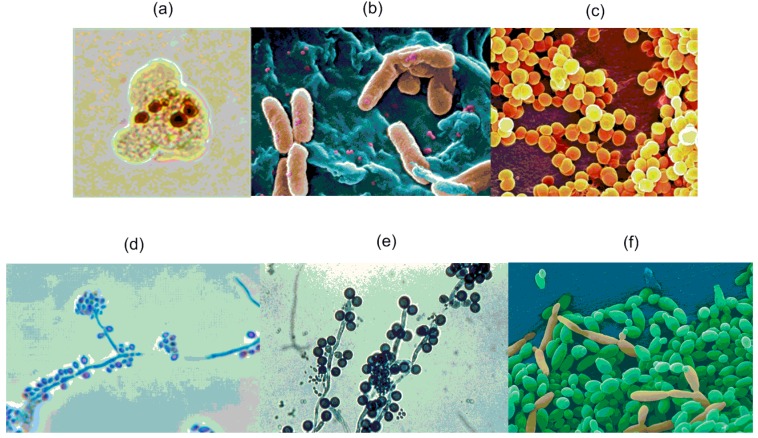
(**a**) *Entamoeba histolytica*; (**b**) *Pseudomonas aeruginosa*; (**c**) *Staphylococcus aureus*, (**d**) *Sporothrix schenckii*; (**e**) *Candida albicans*; and (**f**) *Candida tropicalis* [10].

Parasites are organisms that live with their hosts causing several of the most common human diseases and are responsible for high morbidity and mortality worldwide [1]. Amoebiasis is a human intestinal infection caused by *Entamoeba histolytica* (Figure 1a) characterized by bloody diarrhea, which often leads to death mainly in tropical countries [2].

Bacteria are prokaryotic microorganisms, which can cause death if invasion to bloodstream occurs [3]. *Pseudomonas aeruginosa* (Figure 1b) is a Gram negative pathogenic bacterium which causes several kinds of human diseases, such as pneumonia, as well as infections in urinary tract, lung, dermis, and other organs and tissues [4]. *Staphylococcus aureus* (Figure 1c) is a Gram positive bacterium responsible for a wide range of human infections such as sepsis, meningitis, skin infections, and some of the most common respiratory diseases, such as the chronic sinusitis [5].

There are several fungi that can act as agents of human disease mainly infecting newborns, immuno-compromised patients, and the elderly because they are widespread in the environment. The incidence of infections worldwide by pathogenic fungi has increased considerably during past few years with a dramatic impact in tropical countries [6]. *Sporothrix schenckii* (Figure 1d) is a dimorphic fungus widely distributed in tropical and subtropical zones, which causes the sporothrichosis by traumatic inoculation of contaminated soil, plants, and organic matter [7]. Despite *Candida albicans* (Figure 1e) is known as harmless commensal yeast, it is the most common human fungal pathogen especially to immunologically weak people. It is responsible for painful mucosal infections, vaginitis, oral-pharangeal thrush, and, in some cases, life-threatening bloodstream infections [8]. Moreover, *Candida tropicalis* (Figure 1f) is the most prevalent pathogenic yeast specie of the *Candida*-non-*albicans* group, which has become in a potentially human health problem mainly in tropical zones because it has shown an increasing resistance to the most potent current antifungal drugs, such as the orally available fluconazole [9].

In turn, protozoan parasites and bacteria also have become in serious worldwide health problems due to their ability to resist the current drugs. In this context, the synthesis of novel compounds and the evaluation of their pharmacological properties are always worth investigating.

As a part of our ongoing program, we have developed short and versatile Ugi-azide based synthetic methods toward tetrazole-containing hybrid compounds such as azepinoindolone-tetrazoles [11], tetrahydro-β-carboline-tetrazoles [12], and *bis*-1,5-disubstituted-1*H*-tetrazoles [13]. Recently, we reported the synthesis of fourteen novel chromone-tetrazoles by an Ugi-azide reaction and their *in vitro* evaluation against *Entamoeba histolytica, Giardia lamblia,* and *Trichomonas vaginalis* [14] since it has been reported that chromone [15,16] and tetrazole [17,18] are the core of numerous bioactive products including antimicrobial drugs. Moreover, it has been reported that addition of fluorine atoms into structure of molecules enhances their biological properties such as metabolic stability, lipophilicity, and bioavailability [19]. In this context, we were interested in the synthesis of novel fluorine-containing chromone-tetrazoles and *in vitro* evaluation for their biological properties against pathogenic parasite (*E. histolytica*), pathogenic bacteria (*P. aeruginosa* and *S. aureus*), and pathogenic fungi (*S. schenckii*, *C. albicans*, and *C. tropicalis*).

## 2. Results and Discussion

### 2.1. Chemistry

The fluorine-containing tetrazol-chromones **1o–r** were synthesized by means of an Ugi-azide reaction from the commercially available 6-fluorochromone-3-carboxaldehyde **2** (R = F), anilines **3**, azidotrimethylsilane (**4**) and the isocyanides **5** using InCl_3_ (5% mol) as catalyst, *i*-PrOH [0.5 M] as solvent, in two hours at room temperature. These optimal conditions were previously reported by us to synthesize the tetrazol-chromones **1a–n** [14] (Table 1). The 6-fluorochromone-3-carboxaldehyde (2) was chosen as opposed to other fluoro-substituted chromone-3-carboxaldehydes in basis to results of Diwakar *et al.* [20]*.* Antimicrobial studies to eight newly synthesized chromones-tetrazoles including a 6-fluoro-substituted analog against *S.* *aureus*, *E.* *faecalis*, *S.* *pneumonia*, and *E.* *coli* were conducted and the 6-fluorine-containing chromone-tetrazole was the most active. Good yields were observed for the synthesis of compounds **1o–r** (80%–85%). In fact, we previously detected that the yields were little dependent on structural variations or electronic nature of substituent of the aldehyde and the aniline moieties, together with the low inductive effect coming from the fluorine atom.

**Table 1:** Synthesis of the chromone-tetrazoles **1a–n** ^a^ and the fluorine-containing chromone-tetrazoles **1o–r**.
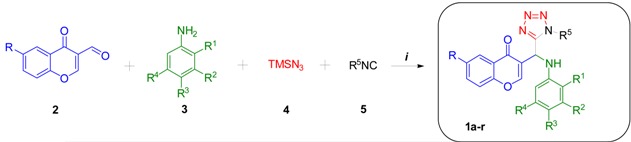

Product	R		R^1^	R^2^	R^3^	R^4^	R^5^	Yield (%) ^b^
****1a**^a^**	H		I	H	H	H	*t*-Bu	85
****1b**^a^**	H		I	H	H	H	Cy	78
****1c**^a^**	H		H	H	H	H	*t*-Bu	86
**1d ^a^**	H		H	H	H	H	Cy	82
**1e ^a^**	H		H	H	Cl	H	*t*-Bu	76
**1f ^a^**	H	H		H	Cl	H	Cy	72
**1g ^a^**	H	H		H	I	H	*t*-Bu	85
**1h ^a^**	H	H		H	I	H	Cy	79
**1i ^a^**	H	H		H	NO_2_	H	Cy	70
**1j ^a^**	H	H		OMe	OMe	H	2,6-MePh	55
**1k ^a^**	H	H		OMe	OMe	OMe	Cy	90
**1l ^a^**	H	Br		H	H	H	*t*-Bu	72
**1m ^a^**	H	Br		H	H	H	Cy	70
**1n ^a^**	H	NO_2_		H	H	H	Cy	71
**1o**	F	H		H	H	H	Cy	81
**1p**	F	H		OMe	OMe	OMe	Cy	85
**1q**	F	H		H	H	H	*t*-Bu	82
**1r**	F	H		H	Cl	H	*t*-Bu	80

^a^ Taken from [14]; ^b^ Measured after recrystallization; *i* = InCl_3_ (5% mol); *i*-PrOH (0.5 M); rt, 2 h.

### 2.2. Biology

#### 2.2.1. Antibacterial and Antiamoebic Activity

In the present study, the series of fourteen previously synthesized chromone-tetrazoles **1a–n** and the four novel fluorine-containing chromone-tetrazoles **1o–r** (Table 1) were tested *in vitro* for their antibacterial activity against *P. aeruginosa*, and *S. aureus*, two pathogenic bacterial strains. Bacterial susceptibility to chromone-tetrazoles was evaluated by determining the Minimal Inhibitory Concentration (MIC) and Minimal Bactericidal Concentration (MBC) (Table 2). The used dilution enabled testing of antibacterial activity of samples, which ranged from 1 to 200 μg/mL. The results indicated that only the **1a**, **1p**, and **1q** compounds showed antibacterial activities, affecting both Gram-positive and Gram-negative bacteria. The other compounds evaluated had not effect in the range of concentrations tested. Among the active compounds, the iodinated **1a** showed activity against Gram-positive bacterium (*S. aureus*) with a MIC of 40 μg/mL, meanwhile the fluorinated **1q** was more active against Gram-negative bacterium (*P. aeruginosa*) with a MIC of 20 μg/mL. A closer structure-activity relationship was observed from the series of chromone-tetrazoles tested. The non-halogenated compounds **1k** and **1c**, without antibacterial activity, have the same basic structure than the fluorinated **1p** and **1q**. In the same way, the iodinated chromone-tetrazole **1a** exhibited antibacterial activity; meanwhile its corresponding non-halogenated chromone-tetrazole **1c** did not. These findings suggest that the antibacterial activity increases when compounds are substituted with fluorine or iodine atoms, while substitution pattern of the tetrazole moiety does not play an essential role.

Previously, we reported the synthesis of the chromone-tetrazoles **1a–n**, which showed *in vitro* activity against the parasite *E. histolytica* [14]. In the present work, we evaluated the antiamoebic effect of the four newly synthesized fluorine-containing chromone-tetrazoles **1o–r** (Table 1). The non-fluorinated compound **1a**, which was tested in the previous work against *Entamoeba histolytica* (IC_50_ = 67.3 μg/mL) [14] was included in the present study. Our results using the compound **1a** exhibited an IC_50_ = 61.7 μg/mL and IC_50_ = 57.1 μg/mL using the microdilution subculture and MTT method, respectively (Table 3). These values are comparable with those obtained in our previous report [14]. The antiamoebic activity of the four fluorine-containing chromone-tetrazoles (**1o–r**) is showed in Table 3. The fluorinated compound **1p** (IC_50_ = 69.5 μg/mL) and the iodinated **1a** (IC_50_ = 61.7 μg/mL) showed a comparable activity against *E. histolytica*, meanwhile the compounds **1o**, **1q**, and **1r** showed low activity (IC_50_ > 200 μg/mL). As it was discussed in our previous work [14], the antiamoebic activity of the chromone-tetrazoles **1a–n** are little dependent on the substitution pattern of chromone, tetrazole, and the aniline moieties.

**Table 2:** Antibacterial activity of chromone-tetrazoles and fluorine-containing chromone-tetrazoles against pathogenic bacterial strains.

Compound	*Pseudomonas aeruginosa*		*Staphylococcus auerus*	
	MIC ^b^	MBC ^b^	MIC ^b^	MBC ^b^
**1a**	160	ND	40	80
**1p**	80	160	160	ND
**1q**	20	40	80	160
Cefotaxime ^a^	0.5	1	1	3

^a^ Antibiotic control; ^b^ MIC and MBC expressed in μg/mL; ND Not determined.

**Table 3:** Half maximal inhibitory concentration of chromone-tetrazoles against *E. histolytica* by Subculture method and MTT assay.

Compound	IC_50_ (μg/mL)	
	Subculture Method	MTT Assay
**1a**	61.7	57.1
**1p**	69.5	71.8
**1q**	228.1	201.7
**1r**	225.1	205.3
**1o**	>320	>320
Metronidazole ^a^	1.5	2.1

^a^ Antiamoebic control.

Hybrid molecules from precursors with known antiparasitic, antifungal and antibacterial activity with azole and azole derivatives showed an increased antimicrobial activity and a broader spectrum of activities [21,22,23]. In the present work, we did not use precursors of well-known antimicrobial activities; instead we tested hybrid molecules with chromone and tetrazole scaffolds, which are cores of numerous bioactive products including antimicrobial drugs [15,16,17,18]. Some of these compounds showed a broad spectrum of activity against pathogenic protozoa, bacteria and fungi. Although these compounds have modest antimicrobial activity, the novelty of this structural class is promising for developing of new antimicrobials.

#### 2.2.2. Antifungal Activity

**Figure 2:**
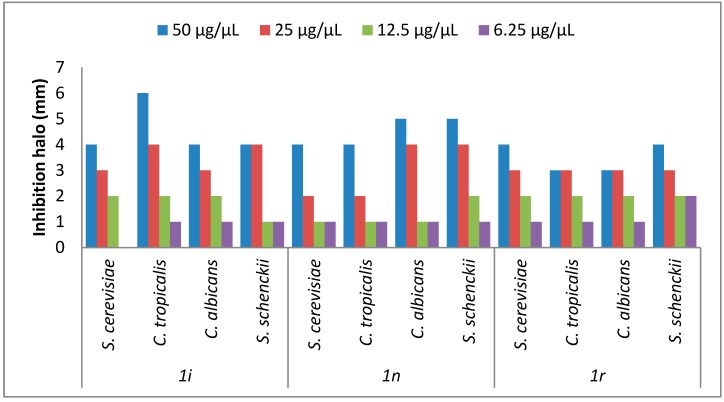
Antifungal Activity of **1i**, **1n**, and **1r** chromone-tretrazoles. The effect of chromone-tetrazoles in solid medium was measured as described in the Experimental Section.

**Figure 3:**
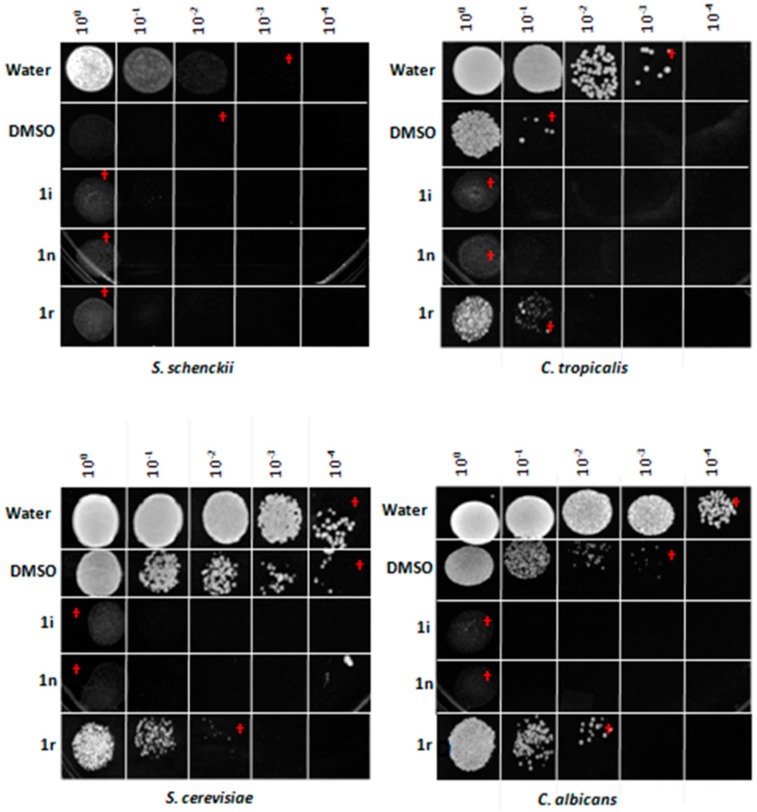
Fungicide Effect of **1i**, **1n**, and **1r** chromone-tetrazoles in liquid medium. The effect of chromone-tetrazoles in liquid medium was measured as described in the Experimental Section. (†) Inhibitory effect on fungal growth observed in solid media.

The antifungal activity from eighteen chromone-tetrazoles was evaluated in both solid and liquid medium. In solid medium, it was determined that just the compounds **1i**, **1n**, and **1r** exhibited antifungal activity at a concentration of 50 µg/µL (See the Supplementary materials for further details). The three compounds shown antifungal activity until a minimum concentration of 6.25 µg/µL with the screened method used (Figure 2). The highest antifungal activity was observed with compound **1r** (Figure 2). Whereas, in liquid culture media all had fungicide effect. The compound **1r** showed a fungistatic effect over *C. tropicalis* at 6.25 µg/µL of test final concentration (Figure 3). The compound **1i** had more effect over *C. tropicalis* growth. Whereas, the compounds **1n** and **1r** had more effect over *S. schenckii* growth. In *S. schenckii*, *C. albicans* and the yeast control *S. cerevisiae* all the compounds had a fungicide effect. In *C. albicans* and *S. cerevisiae* the effect of the compound **1r** was less significative. In *C. tropicalis* the chemicals **1i** and **1n** had fungicide effect, whereas **1r** had an apparent fungistatic effect. The solvent DMSO had no effect in solid medium but it had in broth test. Hazen reported that DMSO has variable responses in growth depending of strain or the *Candida* specie treated. In 1% of DMSO, there is more effect in *C.* *tropicalis* strains than in most of *C.* *albicans* strains as we report here. It could be related to differences in ABC transporters and cell wall proteins between species, as these are involved in organic solvents tolerance in several fungi. DMSO is miscible in water and in combination with higher water activity in liquid media the effect is improved. The basis of this variability is not clear but may reflect the physiology state of the cells at the time of DMSO exposure. Dimethylsulfoxide (DMSO) binds within the plasma membrane of cells and increases membrane permeability and with it the susceptibility to some toxic compounds present in liquid medium, derivated or not from its own metabolism [24].

Compounds **1i**, **1n** and **1r** emerged as the most active congener in the series of synthesized compounds against *C. albicans*, *C. tropicalis* and *S. schenckii* growth. Subsequent experiments to determine Minimal Inhibitory Concentration (MIC) according to the gold standard antifungal test of the Clinical and Laboratory Standards Institute (CLSI), using fluconazole as control drug will help us to define if the above mentioned compounds have potential to be used for clinical purposes, as reported by Attia *et al.*, for certain novel 3-(1*H*-imidazol-1-yl)propan-1-one oxime esthers [25].

## 3. Experimental Section

### 3.1. General Information, Instrumentation, and Chemicals

^1^H- and ^13^C-NMR spectra were acquired on a Bruker Advance III spectrometer (500 MHz, Fällande, Uster, Switzerland). The solvent was CDCl_3_. Chemical shifts are reported in parts per million (δ/ppm). Internal reference for NMR spectra is respect to TMS at 0.0 ppm. Coupling constants are reported in Hertz (*J*/Hz). Multiplicities of the signals are reported using the standard abbreviations: singlet (s), doublet (d), triplet (t), quartet (q), and multiplet (m). NMR spectra were analyzed using the MestreNova software version 6.0.2-5475. IR spectra were recorded on a Perkin Elmer 100 FT-IR spectrometer (Seer Green, Beaconsfield, UK) by the ATR method using neat compounds. The wavelengths are reported in reciprocal centimeters (ν/cm^−1^). HRMS spectra were acquired on a Bruker Daltonics Maxis Impact ESI-QTOF MS (Bremen, Bremen, Germany). HRMS samples were ionized by ESI^+^ mode and recorded via the TOF method. Reaction progress was monitored by TLC, and the spots were visualized under UV light at 254 or 365 nm. Melting points were determined on a Fischer-Johns apparatus (Stone, Staffordshire, UK) and were uncorrected. Cold anhydrous diethyl ether was used as recrystallization solvent. Mixtures of hexanes (Hex) with ethyl acetate (AcOEt) were used to run TLC and for measuring retention factors (R*_f_*). All commercially available starting materials were used without further purification. Chemical names and drawings were obtained using the ChemBioDraw Ultra 13.0.0.3015 software package. The purity for all the synthesized compounds (up to 99%) was assessed by NMR.

### 3.2. General Procedure (GP) for the Synthesis of Fluorine-Containing Chromone-Tetrazoles **1o–r**

To a stirred solution of 6-fluorochromone-3-carboxaldehyde (**2**) (1.0 mmol, 1.0 equiv.) in anhydrous isopropanol [0.5 M] at room temperature, InCl_3_ (5% mol), anilines **3** (1.0 mmol, 1.0 equiv.), azidotrimethylsilane (**4**) (1.0 mmol, 1.0 equiv.) and isocyanides **5** (1.0 mmol, 1.0 equiv) were sequentially added. After stirring for 2 h at room temperature, the solvent was removed until dryness. Then, the crude was dissolved in DCM (20 mL) and washed with a saturated solution of NaHCO_3_ (10 mL) followed by treatment with excess of brine. The organic layer was dried over anhydrous Na_2_SO_4_ and concentrated under vacuum. Finally, the new crude was re-crystallized using cold anhydrous diethylether to afford the products **1o–r** as solids.

### 3.3. Synthesis and Characterization of Fluorine-Containing Chromone-Tetrazoles **1o–r**

#### 3.3.1. 3-((1-Cyclohexyl-1*H*-tetrazol-5-yl)(phenylamino)methyl)-6-fluoro-4*H*-chromen-4-one **1o**

Based on the GP, 6-fluorochromone-3-carboxaldehyde (196.1 mg), aniline (90.0 μL), azidotrimethylsilane (140.0 μL), and cyclohexylisocyanide (128.0 μL) were reacted together in anhydrous *i-*PrOH (2.0 mL) to afford the compound **1o** (335.6 mg, 80%) as a white solid; mp = 241–243 °C; R*_f_* = 0.4 (Hex-AcOEt, 7:3 *v*/*v*); FT-IR (ATR) ν_max_/cm^−1^ 3368 (N–H), 1640 (C=O), 1293 (N–N=N), 1224 (C–O–C); ^1^H-NMR (500 MHz, CDCl_3_, 25 °C): δ 8.30 (s, 1H), 7.82 (dd, *J* = 8.1, 3.1 Hz, 1H), 7.50–7.46 (m, 1H), 7.44–7.39 (m, 1H), 7.22–7.18 (m, 2H), 6.83–6.79 (m, 1H), 6.69 (d, *J* = 7.9 Hz, 2H), 6.15 (d, *J* = 7.7 Hz, 1H), 4.84 (d, *J* = 7.7 Hz, 1H), 4.72–4.64 (m, 1H), 2.10–1.87 (m, 6H), 1.78–1.73 (m, 1H), 1.54–1.38 (m, 2H), 1.36–1.24 (m, 1H); ^13^C-NMR (125 MHz, CDCl_3_, 25 °C): δ 176.1, 176.0, 160.7, 158.8, 155.6, 154.1, 152.7, 144.7, 129.7, 124.6, 124.6, 122.7, 122.5, 120.9, 120.6, 120.5, 119.6, 113.8, 110.6, 110.4, 58.4, 44.5, 33.3, 32.7, 25.3, 24.8; HRMS (ESI^+^) *m*/*z* calcd. for C_23_H_23_FN_5_O_2_^+^ [M + H]^+^ 420.1830; found 420.1853.

#### 3.3.2. 3-((1-Cyclohexyl-1*H*-tetrazol-5-yl)((3,4,5-trimethoxyphenyl)amino)methyl)-6-fluoro-4*H*-chrome-4-one **1p**

Based on the GP, 6-fluorochromone-3-carboxaldehyde (196.1 mg), 3,4,5-trimethoxyaniline (186.9 mg), azidotrimethylsilane (140.0 μL), and cyclohexylisocyanide (128.0 μL) were reacted together in anhydrous *i-*PrOH (2.0 mL) to afford the compound **1p** (433.1 mg, 85%) as a white powder; mp = 227–229 °C; R*_f_* = 0.37 (Hex-AcOEt, 7:3 *v*/*v*); FT-IR (ATR) ν_max_/cm^−1^ 3328 (N–H), 1645 (C=O), 1276 (N–N=N), 1231 (C–O–C); ^1^H-NMR (500 MHz, CDCl_3_, 25 °C): δ 8.32 (s, 1H), 7.83 (dd, *J* = 8.0, 3.1 Hz, 1H), 7.52–7.49 (m, 1H), 7.46–7.42 (m, 1H), 6.17 (d, *J* = 7.6 Hz, 1H), 5.95 (s, 2H), 4.85 (d, *J* = 7.6 Hz, 1H), 4.65–4.59 (m, 1H), 3.77 (s, 6H), 3.74 (s, 3H), 2.01–1.89 (m, 6H), 1.79–1.74 (m, 1H), 1.50–1.40 (m, 2H), 1.35–1.28 (m, 1H); ^13^C-NMR (125 MHz, CDCl_3_, 25 °C): δ 175.9, 160.8, 158.8, 156.1, 154.1, 152.6, 141.5, 131.5, 124.5, 124.4, 122.8, 122.6, 121.3, 120.7, 120.6, 110.5, 110.3, 91.7, 61.0, 58.4, 56.0, 44.5, 33.1, 32.8, 25.3, 25.2, 24.8; HRMS (ESI^+^) *m*/*z* calcd. for C_26_H_29_FN_5_O_5_^+^ [M + H]^+^ 510.2147; found 510.2185.

#### 3.3.3. 3-((1-(*tert*-Butyl)-1*H*-tetrazol-5-yl)(phenylamino)methyl)-6-fluoro-4*H*-chromen-4-one **1q**

Based on the GP, 6-fluorochromone-3-carboxaldehyde (196.1 mg), aniline (90.0 μL), azidotrimethylsilane (140.0 μL), and *tert*-butylisocyanide (120.0 μL) were reacted together in anhydrous *i-*PrOH (2.0 mL) to afford the compound **1q** (318.7 mg, 81%) as a white powder; mp = 179–181 °C; R*_f_* = 0.57 (Hex-AcOEt, 1:1 *v*/*v*); FT-IR (ATR) ν_max_/cm^−1^ 3352 (N-H), 1648 (C=O), 1288 (N–N=N), 1230 (C–O–C); ^1^H-NMR (500 MHz, CDCl_3_, 25 °C): δ 8.30 (s, 1H), 7.80 (dd, *J* = 8.1, 3.1 Hz, 1H), 7.50–7.47 (m, 1H), 7.44–7.39 (m, 1H), 7.23–7.16 (m, 2H), 6.83–6.79 (m, 1H), 6.68 (d, *J* = 7.9 Hz, 2H), 6.47 (d, *J* = 8.2 Hz, 1H), 4.43 (d, *J* = 8.3 Hz, 1H), 1.84 (s, 9H); ^13^C-NMR (125 MHz, CDCl_3_, 25 °C): δ 176.2, 176.1, 160.7, 158.7, 155.8, 154.1, 152.8, 144.6, 129.7, 124.7, 124.6, 122.5, 122.3, 121.2, 120.5, 119.5, 113.4, 110.6, 110.4, 62.3, 45.2, 30.0; HRMS (ESI^+^) *m*/*z* calcd. for C_21_H_21_FN_5_O_2_^+^ [M + H]^+^ 394.1673; found 394.1684.

#### 3.3.4. 3-((1-(*tert*-Butyl)-1*H*-tetrazol-5-yl)((4-chlorophenyl)amino)methyl)-6-fluoro-4*H*-chromen-4-one **1r**

Based on the GP, 6-fluorochromone-3-carboxaldehyde (196.0 mg), 4-chloroaniline (130.0 mg), azidotrimethylsilane (140.0 μL), and *tert*-butylisocyanide (120.0 μL) were reacted together in anhydrous *i-*PrOH (2.0 mL) to afford the compound **1r** (342.2 mg, 80%) as a white solid; mp = 226–228 °C; R*_f_* = 0.34 (Hex-AcOEt, 7:3 *v*/*v*); FT-IR (ATR) ν_max_/cm^−1^ 3310 (N–H), 1642 (C=O), 1280 (N–N=N), 1221 (C–O–C); ^1^H-NMR (500 MHz, CDCl_3_, 25 °C): δ 8.29 (s, 1H), 7.80 (dd, *J* = 8.1, 3.0 Hz, 1H), 7.51–7.48 (m, 1H), 7.45–7.40 (m, 1H), 7.13 (d, *J* = 8.8 Hz, 2H), 6.62 (d, *J* = 8.8 Hz, 2H), 6.42 (d, *J* = 8.4 Hz, 1H), 4.69 (d, *J* = 8.4 Hz, 1H), 1.83 (s, 9H); ^13^C-NMR (125 MHz, CDCl_3_, 25 °C): δ 176.1, 160.7, 158.7, 155.9, 153.7, 152.7, 143.4, 129.5, 124.6, 124.5, 124.1, 122.6, 122.4, 120.8, 120.5, 114.5, 110.5, 110.3, 62.4, 45.3, 29.9; HRMS (ESI^+^) *m*/*z* calcd. for C_21_H_20_ClFN_5_O_2_^+^ [M + H]^+^ 428.1284; found 428.1296.

### 3.4. Antibacterial Assay

The antibacterial activity of the synthesized chromone-tetrazoles was assessed by employing a standard dilution method, determining the minimum inhibitory concentration (MIC) and minimum bactericidal concentration (MBC) [26]. Glass tubes were used for the tests. The assay was carried out with two bacterial species, the Gram-negative bacterium *Pseudomonas aeruginosa* ATCC 13384 and the Gram-positive bacterium *Staphylococcus aureus* ATCC 6538. The inoculum was an overnight culture of each bacterial species in LB broth diluted in the same media to a final concentration of 100 CFU/mL. The chromone-tetrazoles were dissolved in dimethyl sulfoxide (DMSO) to a concentration of 20 mg/mL. Further dilutions were performed in LB broth containing each bacteria species to reach a final concentration range of 10 to 160 μg/mL. MIC was determined after incubation at 36 °C for 16 h. Bacterial growth was detected by optical density determination at 600 nm (Spectrophotometer GeneQuant-pro Amersham). The MBC was examined by plating dilutions of the antimicrobial concentrations that inhibited the visible growth of the bacterium on LB-agar plates. MIC and MBC values were defined as the lowest concentration of each chromone-tetrazole tested, which completely inhibited growth or yielded no viable microorganisms, respectively. The antibiotic cefotaxime (Sigma-Aldrich, St. Louis, MO, USA) was used as control bactericidal tests of the reference strains [26].

### 3.5. Antiamoebic Assay

The virulent strain of *Entamoeba histolytica* (HM-1-IMSS) was grown axenically at 37 °C in TYI-S-33 medium supplemented with 10% heat inactivated bovine serum [27]. Trophozoites at the exponential phase of growth were used in all experiments. Antiamoebic activity of the synthesized chromone-tetrazoles in DMSO was assessed using the standard dilution micromethod [28,29] and subculture [30]. We introduced 2 × 10^4^ trophozoites in 100 μL of TYI-S-33 per each well of the 96 well microtiter plate (Nunc Thermo Scientific) and allow the parasites to adhere at the bottom of the well at 37 °C for 2 h, then 100 μL of different concentrations (10–320 μg/mL) of each chromone-tetrazole tested in TYI-S-33 was added and incubated at 37 °C for 24 h. The trophozoites were detached by chilling at 4 °C for 10 min and transferred to new culture tubes with fresh medium without antibiotic and incubated for 48 h at 37 °C. The final number of parasites was determined with a hemocytometer and the percentages of growth inhibition were calculated by comparison with the control culture. Each test included metronidazole (U.S.P. Poulenc, Lt. Montreal, QC, Canada) as standard amoebicidal drug and a control containing DMSO. The concentration of DMSO did not exceed 1% in all assays performed. Each assay was performed in duplicated and repeated three times. The antiamoebic activity was also evaluated by MTT assay [31]. After incubation of parasites with chromone-tetrazoles as described above, the trophozoites were washed and incubated for 45 min at 37 °C in a saline phosphate buffer with 0.5 mg/mL of MTT (3-[4,5-dimethylhiazol-2-il]-2,5-diphenyl tetrazolium bromide) (Sigma). The formazan crystals produced by dye were extracted with 10% Triton X-100 plus 0.1 N HCl in *i*-PrOH and the absorbance was determined at two different wavelengths (540 nm and 690 nm) by using a microplate reader (Multiskan Go Thermo Scientific, Vantaa, Helsinki, Finland). The experiments were performed by duplicate and repeated three times.

### 3.6. Antifungal Assay

The antifungal activity against human pathogenic fungi was evaluated in solid medium by antibiotic susceptibility testing using disc diffusion method [32]. The protocol summarizes as follows, the fungi were grown in 5 mL of YPD broth by 24 h for *Candida* species and *Sacharomyces cerevisiae*, and by 48 h for *Sporothrix schenckii* yeast; then, the cell density was adjusted at absorbance 1 by spectrophotometric reading at 600 nm. One hundred microliters of cell suspension was inoculated over YPD plates. Sterile paper discs of 6 mm in diameter were soaked with 10 μL of each chemical solution and deposited over previously inoculated agar surface. Petri dishes were incubated at 24 °C for 48 h. We used DMSO as control. The antifungal activity of the **1i**, **1n**, and **1r** chromone-tetrazoles was evaluated in liquid medium as follows, the fungi were grown in 5 mL of YPD broth by 24 h for *Sacharomyces cerevisiae* and *Candida* species or in YPD pH 7.8 by 72 h for *Sporothrix schenckii*, all incubated at 37 °C to get yeast morphology. Yeast cells were accounted in a Neubauer chamber. Then, 2 × 10^6^ cells were inoculated in eppendorf tubes with 100 µL of liquid medium. YPD pH 7.8 broth for *S. schenckii* or YPD broth for the other fungal species, whit a final concentration of 6.25 µg/µL of each compound tested, then the tubes were incubated for 24 h at 37 °C and 150 rpm agitation. After this time, every cell suspension were diluted with sterile water to get dilutions until 1 × 10^−4^, 10 µL of all dilutions were inoculated in order over YPD plates and incubated 24 h at 37 °C. Controls were DMSO and sterile water.

## 4. Conclusions

Four novel fluorine-containing chromone-tetrazoles were quickly synthesized in good yields (80–85) by means of an Ugi-azide reaction using our previously reported optimal conditions. Compounds **1a** and **1p** are the most promising molecules since they show *in vitro* activity against both protozoa and bacteria as compared to other tested compounds. In solid culture medium, all compounds had activity at the minimum concentration tested. The compound **1i** had more effect over *C. tropicalis*, whereas, compounds **1n** and **1r** had more effect over *S. schenckii*. In liquid culture medium, compounds **1i**, **1n** and **1r** had fungicide activity over *S. schenckii*, *C. albicans* and the yeast control *S. cerevisiae*.With *C. albicans* and *S. cerevisiae* compound **1n**, the effect was less significative. In *C. tropicalis*, the compounds **1i** and **1n** had fungicide effect, whereas product **1r** had a fungistatic effect. As can be seen, the values of MIC indicate moderate sensitivity of bacteria and fungi. In the same context, the IC_50_ values indicate moderate sensitivity of *E. histolytica*. However, due to the resistance of microbes to the current drugs, the synthesis and study of novel antimicrobial compounds is necessary.

## Supplementary Materials

Supplementary materials can be accessed at: http://www.mdpi.com/1420-3049/20/07/12436/s1.

## References

[1] Gillespie S.H., Pearson R.D. (2001). Principles and Practice of Clinical Parasitology.

[2] Skappak C., Akierman S., Belga S., Novak K., Chadee K., Urbanski S. J., Church D., Beck P.L. (2014). Invasive amoebiasis: A review of *Entamoeba* infections highlighted with case reports. Can. J. Gastroenterol. Hepatol..

[3] Koch A.L. (2006). Bacterial Morphologies. The Bacteria: Their Origin, Structure, Function and Antibiosis.

[4] Strateva T., Yordanov D. (2009). *Pseudomonas aeruginosa*—A phenomenon of bacterial resistance. J. Med. Microbiol..

[5] Plata K., Rosato A.E., Wegrzyn G. (2009). *Staphylococcus aureus* as an infectious agent: Overview of biochemistry and molecular genetics of its pathogenicity. Acta Biochim. Pol..

[6] Hazen K.C. (1995). New and emerging yeast pathogens. Clin. Microbiol. Rev..

[7] Barros M.B.D.L., de Almeida Paes R., Schubach A.O. (2011). *Sporothrix schenckii* and Sporotrichosis. Clin. Microbiol. Rev..

[8] Kim J., Sudbery P. (2011). *Candida albicans*, a major human fungal pathogen. J. Microbiol..

[9] Kothavade R.J., Kura M.M., Valand A.G., Panthaki M.H. (2010). *Candida tropicalis*: Its prevalence, pathogenicity and increasing resistance to fluconazole. J. Med. Microbiol..

[B10-molecules-20-12436] 10.The images described in Figure 1 are of public domain or protected by the rights indicated: For (a) the *E. histolytica* image, author: Felipe Padilla-Vaca (F.P.-V.); (b) *Pseudomonas aeruginosa* image, ID number: #10043, Centers of Disease Control and Prevention’s, CDC; (c) *Staphylococcus aureus*, ID number: C010/5904, Science Photo Library; (d) *Sporothrix schenckii*, ID number: 4208, Centers of Disease Control and Prevention’s, CDC; (e) *Candida albicans* image, ID: “*Chlamydospores of Candida albicans*” author: Goodman, on microbewolrd.org; (f) *Candida tropicalis* image, shared with free license of Creative Commons

[11] Gordillo-Cruz R.E., Rentería-Gómez A., Islas-Jácome A., Cortes-García C.J., Díaz-Cervantes E., Robles J., Gámez-Montaño R. (2013). Synthesis of 3-tetrazolylmethyl-azepino[4,5-*b*]indol-4-ones in two reaction steps: (Ugi-azide/*N*-acylation/S_N_2)/free radical cyclization and docking studies to a 5-Ht_6_ model. Org. Biomol. Chem..

[12] Cárdenas-Galindo L.E., Islas-Jácome A., Alvarez-Rodríguez N.V., El Kaim L., Gámez-Montaño R. (2014). Synthesis of 2-Tetrazolylmethyl-2,3,4,9-tetrahydro-1*H*-β-carbolines by a One Pot Ugi-azide/Pictet-Spengler Process. Synthesis.

[13] Cárdenas-Galindo L.E., Islas-Jácome A., Colmenero-Martínez K.M., Martínez-Richa A., Gámez-Montaño R. (2015). Synthesis of Novel *bis*-1,5-Disubstituted-1*H*-Tetrazoles by an Efficient Catalyst-Free Ugi-azide Repetitive Process. Molecules.

[14] Cano P.A., Islas-Jácome A., González-Marrero J., Yépez-Mulia L., Calzada F., Gámez-Montaño R. (2014). Synthesis of 3-tetrazolylmethyl-4*H*-chromen-4-ones via Ugi-azide and biological evaluation against *Entamoeba histolytica*, *Giardia lamblia* and *Trichomonas vaginalis*. Bioorg. Med. Chem..

[15] Gaspar A., Matos M.J., Garrido J., Uriarte E., Borges F. (2014). Chromone: A Valid Scaffold in Medicinal Chemistry. Chem. Rev..

[16] Khadem S., Marles R.J. (2012). Chromone and Flavonoid Alkaloids: Occurrence and Bioactivity. Molecules.

[17] Sarvary A., Maleki A. (2014). A review of syntheses of 1,5-disubstituted tetrazole derivatives. Mol. Divers..

[18] Myznikov L.V., Hrabalek A., Koldobskii G.I. (2007). Drugs in the tetrazole series. Chem. Heterocycl. Comp..

[19] Purser S., Moore P.R., Swallow S., Gouverneur V. (2008). Fluorine in medicinal chemistry. Chem. Soc. Rev..

[20] Diwakar S.D., Bhagwat S.S., Shingare M.S., Gill C.H. (2008). Substituted 3-((*Z*)-2-(4-nitrophenyl)-2-(1*H*-tetrazol-5-yl) vinyl)-4*H*-chromen-4-ones as novel anti-MRSA agents: Synthesis, SAR, and *in vitro* assessment. Bioorg. Med. Chem. Lett..

[21] Saadeh H.A., Mosleh I.M. I.M., Mubarak M.S. (2009). Synthesis of Novel Hybrid Molecules from Precursors with Known Antiparasitic Activity. Molecules.

[22] Wang Y., Damu G.L.V., Lv J.S., Geng R.X., Yang D.C., Zhou C.H. (2012). Design, synthesis and evaluation of clinafloxacin triazole hybrids as a new type of antibacterial and antifungal agents. Bioorg. Med. Chem. Lett..

[23] Negi B., Raj K.K., Siddiqui S.M., Ramachandran D., Azam A., Rawat D.S. (2014). *In Vitro* Antiamoebic Activity Evaluation and Docking Studies of Metronidazole-Triazole Hybrids. Chem. Med. Chem..

[24] Hazen K.C. (2013). Influence of DMSO on antifungal activity during susceptibility testing *in vitro*. Diagn. Microbiol. Infect. Dis..

[25] Attia M.I., Zakaria A.S, Almutairi M.S., Ghoneim S.W. (2013). *In Vitro* Anti-*Candida* Activity of Certain New 3-(1*H*-Imidazol-1-y1)propan-1-one oxime Esters. Molecules.

[26] Andrews J.M. (2001). Determination of minimum inhibitory concentrations. J. Antimicrob. Chemother..

[27] Diamond L.S., Harlow D.R., Cunnick C.C. (1978). A new medium for the axenic cultivation of *Entamoeba histolytica* and other *Entamoeba*. Trans. R. Soc. Trop. Med. Hyg..

[28] Wright C.W., O’Neill M.J., Phillipson J.D., Warhurst D.C. (1988). Use of microdilution to assess *in vitro* antiamoebic activities of *Brucea javanica* fruits, *Simarouba amara* stem, and a number of quassinoids. Antimicrob. Agents Chemother..

[29] Upcroft J.A., Upcroft P. (2001). Drug Susceptibility Testing of Anaerobic Protozoa. Antimicrob. Agents Chemother..

[30] Calzada F., Meckes M., Cedillo-Rivera R., Tapia-Contreras A., Mata R. (1998). Screening of Mexican medicinal plants for antiprotozoal activity. Pharm. Biol..

[31] Kodama E., Igarashi A., Mori S., Hashimoto K.I., Suzuki T., DeClercq E., Shigeta S. (1996). Evaluation of antiherpetic compounds using a gastric cancer cell line: Pronounced activity of BVDU against herpes simplex virus replication. Microbiol. Immunol..

[32] Bauer A.W., Kirby W.M., Sherris J.C., Turk M. (1966). Antibiotic susceptibility testing by a standardized single disk method. Am. J. Clin. Pathol..

