# Supplementary material for: In Vitro Studies of Chromone-Tetrazoles against Pathogenic Protozoa, Bacteria, and Fungi
Source: Molecules. 2015 Jul 8;20(7):12436–49. doi: 10.3390/molecules200712436 (PMC6332438; doi:10.3390/molecules200712436)
Supplement: Supplementary file 1 [file molecules-20-12436-s001.pdf]

Supplementary File

Content

NMR Spectra of compounds **1o–r** S1

Antifungal activity S5

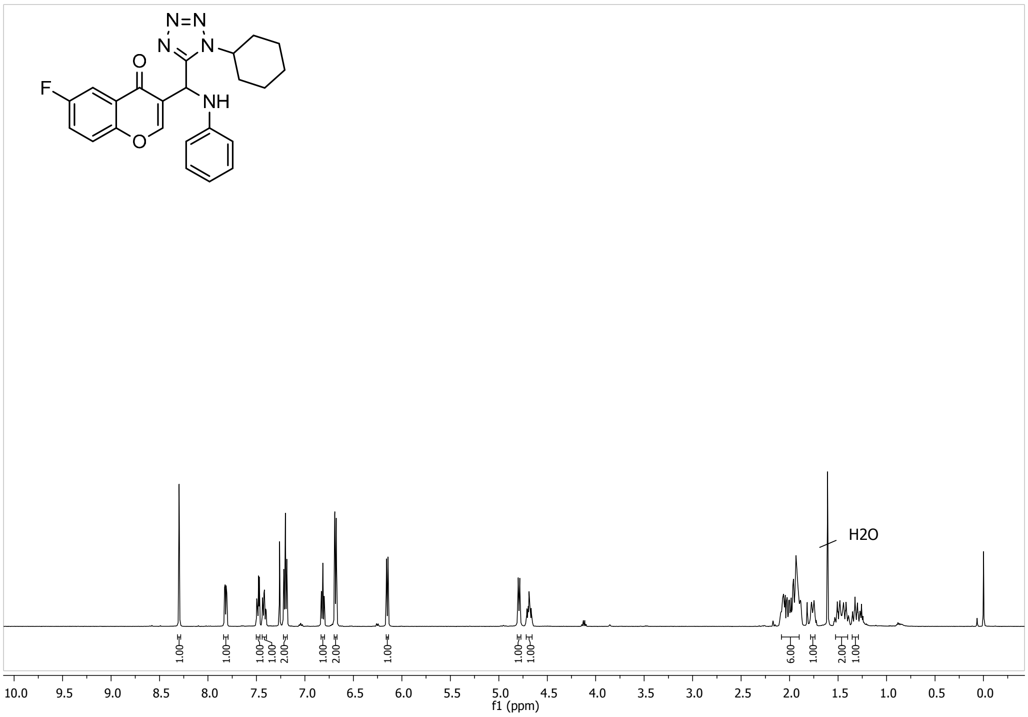

Figure S1. ^{1}H-NMR spectra of compound **1o**.

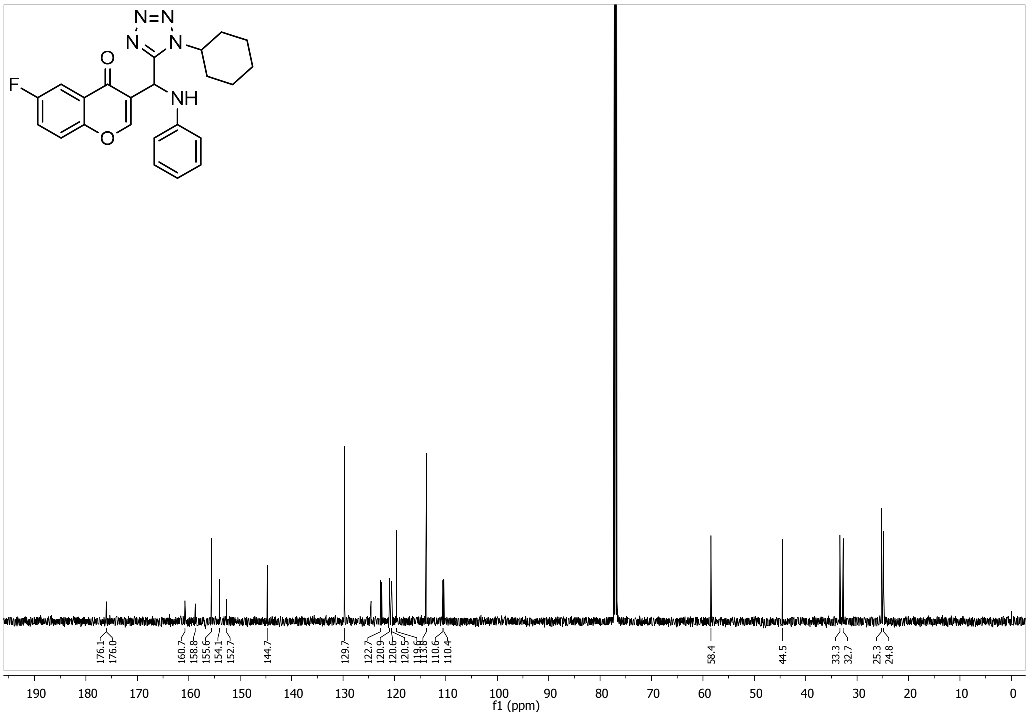

Figure S2. ^{13}C-NMR spectra of compound **1o**.

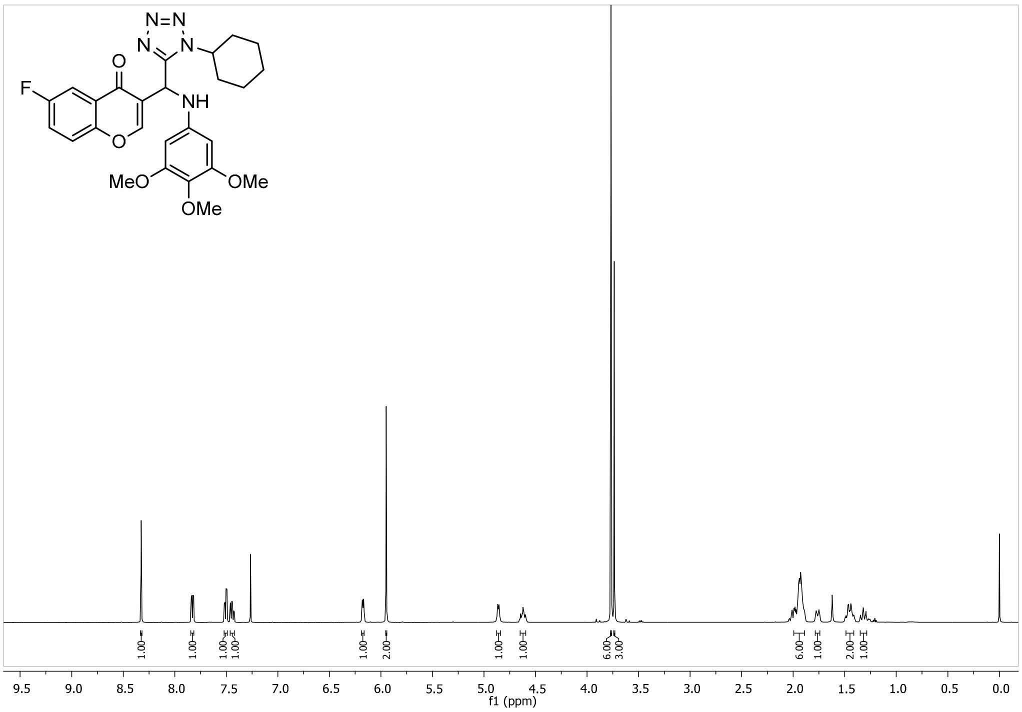

Figure S3. ^{1}H-NMR spectra of compound **1p**.

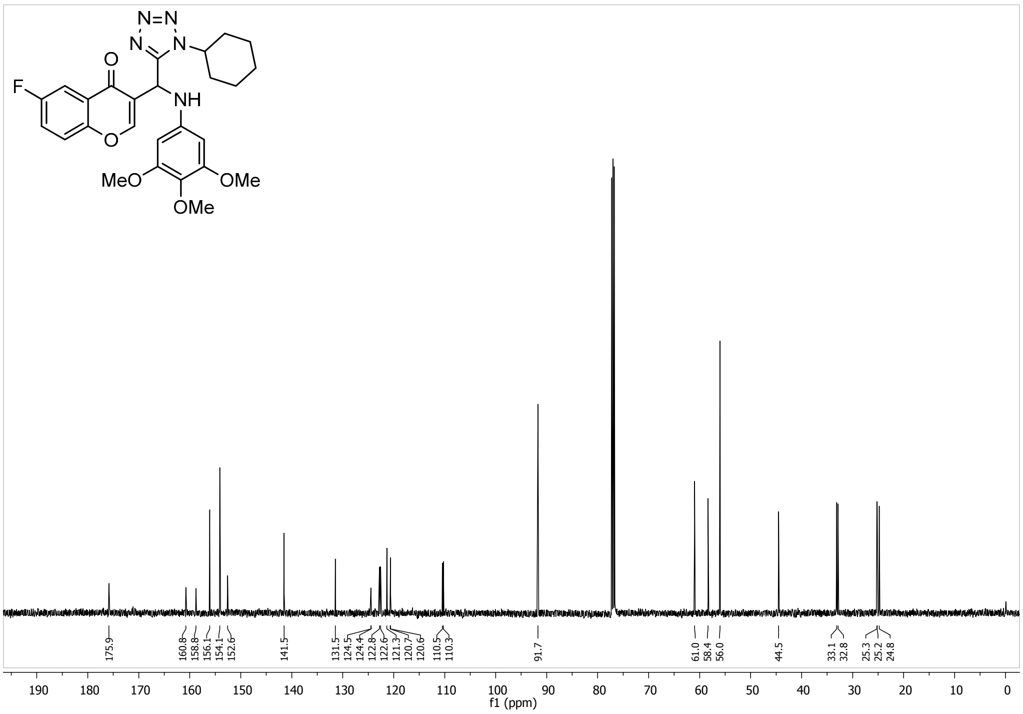

Figure S4. ^{13}C-NMR spectra of compound **1p**.

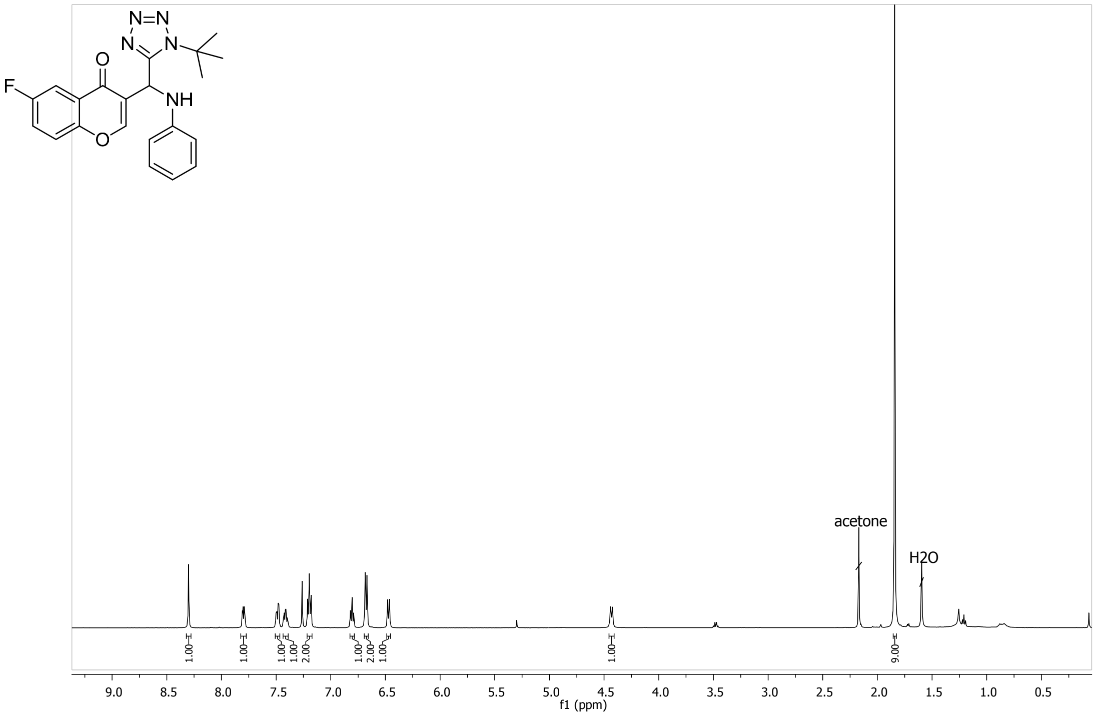

Figure S5. ^{1}H-NMR spectra of compound **1q**.

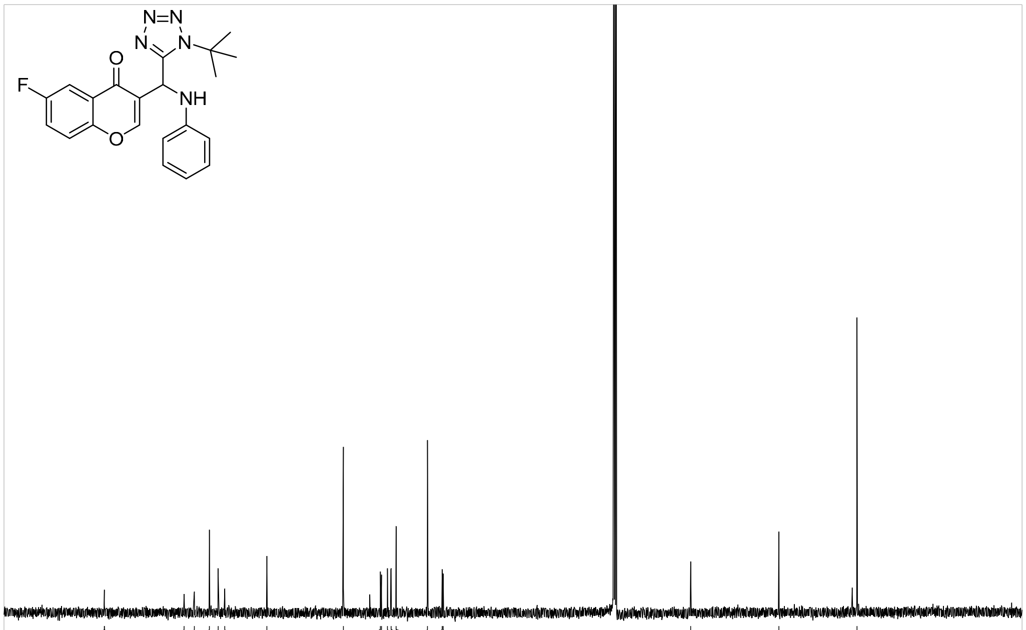

Figure S6. ^{13}C-NMR spectra of compound **1q**.

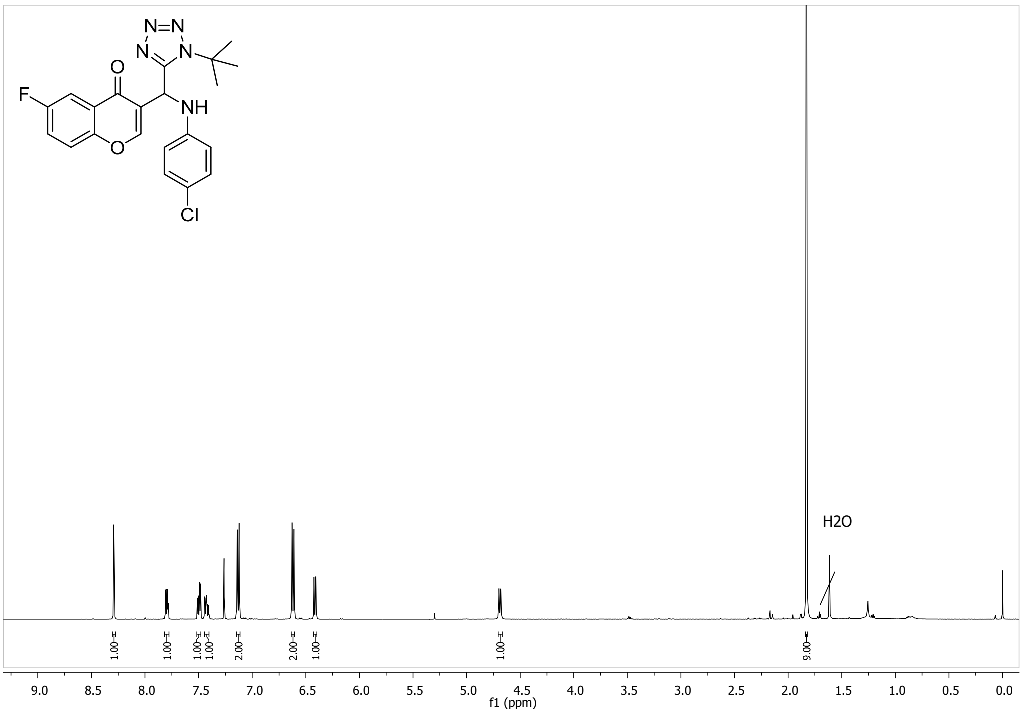

Figure S7. ^{1}H-NMR spectra of compound **1r**.

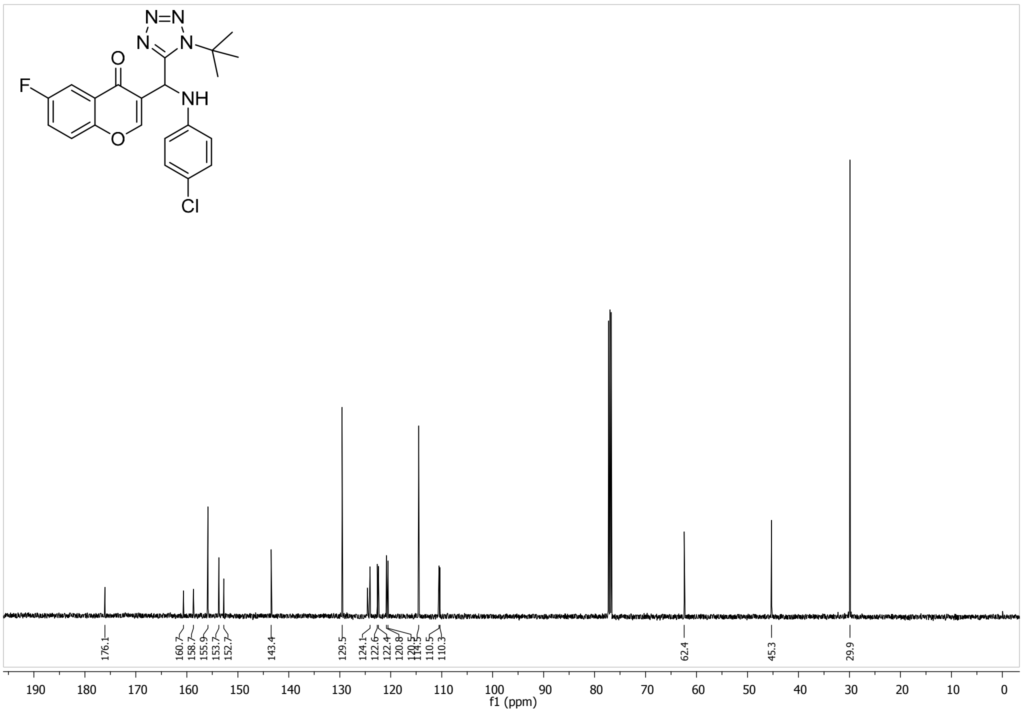

Figure S8. ^{13}C-NMR spectra of compound **1r**.

Antifungal activity

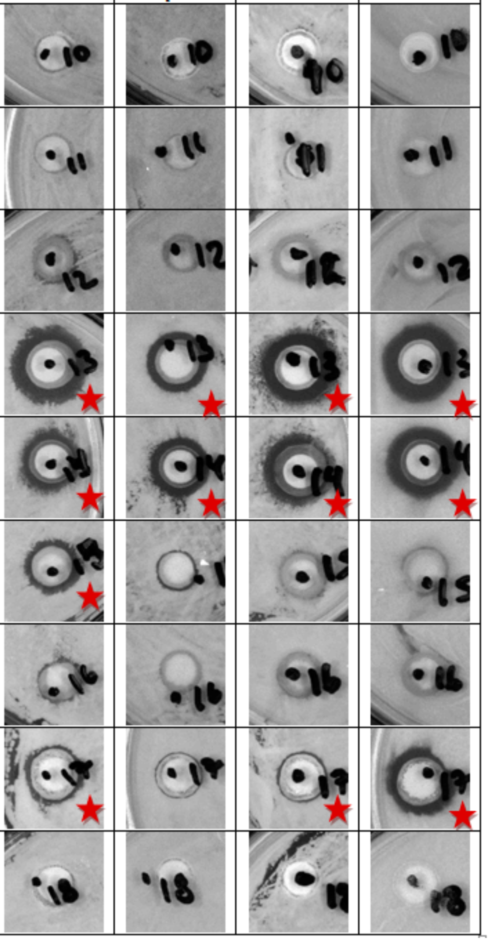

	S. cerevisiae	*C. tropicalis*	*C. albicans*	*S. schenckii*
Compound 1m				
Compound 1j				
Compound 1k				
Compound 1i	★	★	★	★
Compound 1n	★	★	★	★
Compound 1p	★			
Compound 1q				
Compound 1r	★		★	★
Compound1o				

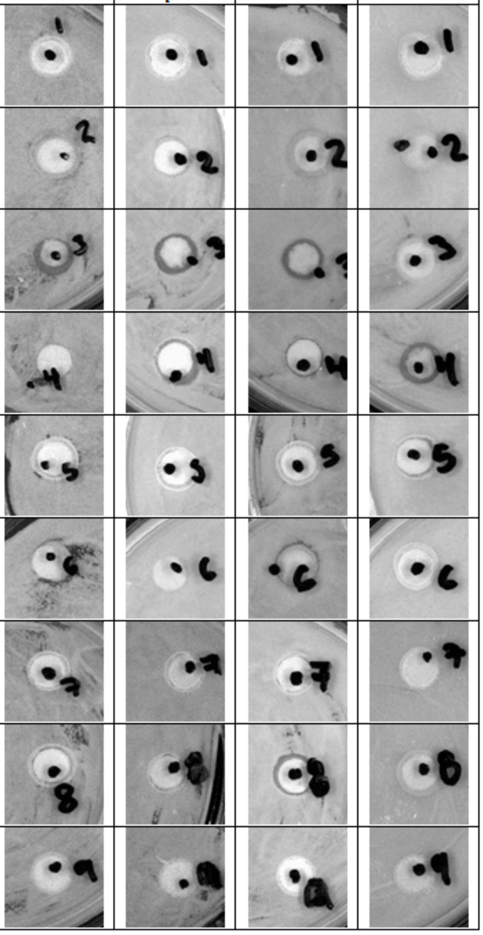

	S. cerevisiae	*C. tropicalis*	*C. albicans*	*S. schenckii*
Compound 1a				
Compound 1b				
Compound 1c				
Compound 1d				
Compound 1e				
Compound 1f				
Compound 1g				
Compound 1h				
Compound 1l				

★ **Fungal growth in solid media.** Significative inhibition with 10 µL of the chromone-tetrazoles solution with a concentration of 50 µg/µL in DMSO.
